# Comparable effectiveness of intensive intravenous and less intensive immunosuppressive treatment in retroperitoneal fibrosis: a retrospective real-world cohort study

**DOI:** 10.1007/s00296-026-06166-5

**Published:** 2026-05-30

**Authors:** Adrianna Niksińska, Robert Kruszewski, Michał Wąsik, Marta Kania-Pudło, Bartłomiej Kisiel, Ewa Malczuk, Jerzy Koblowski, Witold Tłustochowicz

**Affiliations:** 1 Department of Internal Medicine and Rheumatology, Military Institute of Medicine, National Research Institute, Warsaw, Poland; 2 Department of Radiology, Military Institute of Medicine, National Research Institute, Warsaw, Poland

**Keywords:** Retroperitoneal fibrosis, Immunosuppressive agents, Renal outcome, Glucocorticoids, Rituximab, Cohort study

## Abstract

Retroperitoneal fibrosis (RPF) is a rare fibro-inflammatory disease frequently complicated by ureteral obstruction and renal impairment. Due to its rarity, heterogeneous clinical course, and the absence of standardized treatment guidelines, optimal immunosuppressive management of RPF remains uncertain. This study aimed to evaluate the real-world effectiveness and safety of different immunosuppressive treatment strategies in patients with RPF. A retrospective, single-center observational study was conducted including patients with idiopathic or immune-mediated RPF treated between 2010 and 2025. Patients were grouped according to treatment strategy: intensive intravenous therapy with cyclophosphamide or rituximab, or less intensive oral immunosuppressive therapy including methotrexate, mycophenolate mofetil or azathioprine. Treatment effectiveness was assessed using a composite endpoint incorporating clinical, laboratory and radiological response. Secondary outcomes included renal function, relapse, treatment safety, and glucocorticoid exposure. Twenty-nine patients were included in the analysis, of whom 11 received intensive therapy and 18 were treated with less intensive oral immunosuppressive regimens. Overall treatment effectiveness was achieved in 68% of evaluable patients and did not differ significantly between groups (OR 2.67, 95% CI 0.41–17.17). Clinical, laboratory, and radiological responses did not differ significantly between treatment groups. Hydronephrosis resolved in 75% of affected patients. Median serum creatinine levels improved during follow-up in both treatment groups, without significant intergroup differences at last observation (1.20 mg/dL [1.10–1.30] vs. 1.20 mg/dL [0.80–1.40]). The median relative reduction in periaortic mass thickness was 41.2% (24.45–53.95). No significant differences in overall safety outcomes were observed. In this real-world cohort of patients with RPF, intensive intravenous immunosuppressive regimens were not associated with superior treatment effectiveness compared with less intensive oral therapies. These findings support an individualized approach to treatment selection in RPF, taking disease severity, renal involvement and patient-specific factors into account. Prospective studies in larger cohorts are needed to further define optimal long-term management strategies for this rare disease.

## Introduction

Retroperitoneal fibrosis (RPF) is a rare disease characterized by the development of fibro-inflammatory tissue surrounding the abdominal aorta and frequently extending to the iliac arteries. Progressive expansion of the retroperitoneal mass may lead to severe complications, most notably ureteral obstruction, resulting in hydronephrosis and renal failure [1, 2]. 

RPF may occur secondary to infections, malignancies, exposure to certain drugs or asbestos, and in association with immune-mediated conditions including connective tissue diseases and IgG4-related disease (IgG4-RD).

In the majority of cases, the cause remains unknown, and the condition is then classified as idiopathic retroperitoneal fibrosis (IRF). The pathogenesis of RPF remains incompletely understood and the disease course is heterogeneous, ranging from indolent disease to aggressive forms requiring prolonged immunosuppressive therapy [3, 4]. 

Chronic periaortitis is a broad term encompassing fibro-inflammatory conditions of the aorta and periaortic tissues, including RPF [2]. 

Clinical manifestations of RPF are nonspecific, including abdominal and lumbar pain, fatigue, weight loss and features of renal impairment. Disease assessment relies on clinical evaluation, inflammatory markers and imaging studies, with contrast-enhanced computed tomography as the primary diagnostic and monitoring tool. Magnetic resonance imaging and positron emission tomography (PET) are used more selectively in specific clinical contexts [2, 4]. However, the most recent studies suggest a possible advantage of PET over computed tomography in assessing metabolically active periaortic infiltrates [5]. 

Pharmacological treatment represents the cornerstone of RPF management and is most commonly initiated with systemic corticosteroids. Despite their established efficacy, there are no guidelines regarding initial dosing or treatment duration, and frequent relapses following glucocorticoid tapering underscore the need for additional immunosuppressive therapy [6, 7, 8]. Conventional immunosuppressive agents, including mycophenolate mofetil (MMF), azathioprine (AZA), methotrexate (MTX) and cyclophosphamide (CYC), have been used with variable results [9–14]. 

An alternative therapeutic option is tamoxifen, although in recent studies the superiority of corticosteroids has been demonstrated [15, 16]. More recently, biologic agents such as rituximab (RTX) and tocilizumab have emerged as potential therapeutic options [17, 18, 19]. 

The choice of immunosuppressive strategy is guided by disease severity, renal involvement, and physician experience.

The definition of treatment efficacy in RPF remains poorly standardized. Therapeutic response has been variably assessed using clinical improvement, reduction of inflammatory markers levels, improvement in renal function, decrease in periaortic soft tissue mass and resolution of hydronephrosis. Among these, preservation of renal function is unquestionably the most clinically relevant outcome; however, published studies differ substantially in the endpoints used to define treatment success [7, 11, 17]. While some reports focus predominantly on radiological outcomes, others adopt composite definitions incorporating clinical, laboratory and imaging responses [10, 13]. In addition, the magnitude of fibrotic mass reduction considered clinically meaningful varies across reports, with authors defining a reduction of at least 20–30% as clinically meaningful [9, 19]. 

Due to the rarity of RPF, available studies are limited by small patient cohorts and predominantly retrospective designs [10, 11, 12]. The effectiveness of various immunosuppressive agents has been proven; however, assembling groups large enough to allow direct comparisons of therapy efficacy remains challenging. These limitations have contributed to the lack of established treatment guidelines of RPF. Furthermore, the disease requires long-term patient monitoring, as an initial therapeutic response does not preclude relapse [11, 13]. Consequently, observational studies with extended follow-up periods provide valuable insights into disease course and treatment outcomes.

Therefore, the aim of this retrospective, single-center, observational study was to compare the effectiveness and safety of intensive intravenous versus less intensive immunosuppressive treatment strategies in patients with RPF with long follow-up period and to evaluate their impact on clinical, renal and radiological outcomes.

## Methods

This was a retrospective, single-center observational cohort study including patients diagnosed with RPF treated at the Department of Internal Medicine and Rheumatology, Military Institute of Medicine, Poland between 2010 and 2025. Medical records were reviewed to collect clinical, laboratory, imaging, and treatment-related data. Follow-up duration was defined as the time from initiation of immunosuppressive therapy to the last documented visit. The duration of follow-up varied between patients and treatment groups.

The diagnosis of RPF was established based on clinical presentation and characteristic findings on contrast-enhanced computed tomography including periaortic retroperitoneal mass and associated hydronephrosis.

Patients were assessed with regard to clinical features, baseline demographic characteristics, laboratory parameters and imaging. The main variables of interest included treatment strategy, presence of hydronephrosis, inflammatory markers, including erythrocyte sedimentation rate (ESR), C-reactive protein (CRP), serum creatinine levels and periaortic mass thickness at baseline and during follow-up. Clinical, laboratory, and radiological outcomes were assessed during follow-up visits as part of routine clinical practice.

Radiological evaluation was performed by two designated radiologists using contrast-enhanced computed tomography. The thickness of the retroperitoneal mass was measured in the axial plane at the site of maximal periaortic involvement at baseline and on follow-up imaging, according to a predefined measurement approach. Radiological response could be assessed only in patients with available baseline and follow-up computed tomography or in patients with resolution of hydronephrosis.

Inclusion criteria comprised adult patients (≥ 18 years) with a diagnosis of RPF who received immunosuppressive therapy during the study period and had at least 3 months of follow-up available after treatment initiation. Both idiopathic and secondary forms of RPF associated with immune-mediated diseases were included.

Exclusion criteria comprised RPF secondary to malignancy, lack of immunosuppressive treatment initiation, and insufficient follow-up data preventing assessment of treatment response.

Patients were divided into two groups according to the intensity and route of immunosuppressive treatment.

Treatment allocation was not randomized and reflected routine clinical practice. Decisions regarding treatment intensity were made by the treating physicians based on individual clinical judgement, taking into account overall patient condition, renal function, presence of hydronephrosis and periaortic mass thickness, reflecting real-world clinical practice.

The intensive treatment group included patients treated with intravenous cyclophosphamide or rituximab. Cyclophosphamide was administered intravenously in intermittent pulses, with a median dose of 6.6 g (2.85–7.35), while patients treated with rituximab received two intravenous infusions of 1000 mg given two weeks apart and two of them received maintenance therapy. The less intensive treatment group comprised patients treated with oral immunosuppressive agents, including methotrexate with a median dose of 20 mg/week (15–25), azathioprine with a median dose of 150 mg/day (100–200), or mycophenolate mofetil with a median dose of 2.5 g/day (2–3). Systemic glucocorticoids were used concomitantly in the majority of patients in both groups, usually as initial therapy, with a median dose of 60 mg/day (30–60) followed by gradual tapering. One patient received glucocorticoid monotherapy without additional immunosuppressive agents. The heterogeneity of immunosuppressive regimens reflects current clinical practice in the absence of standardized treatment guidelines for RPF.

Therapy duration and dose adjustments were individualized based on clinical response, laboratory results, and imaging findings.

The primary outcome of the study was treatment effectiveness, while secondary outcomes included renal outcome, safety of the treatment, mortality and disease relapses.

Treatment was considered effective if all three components of response were met. These included absence of clinical symptoms during follow-up, laboratory improvement defined as normalization or a ≥ 50% decrease in CRP and ESR levels, and radiological response classified as a reduction in the thickness of the periaortic retroperitoneal mass by at least 20% or resolution of hydronephrosis on follow-up imaging.

All components had to be fulfilled simultaneously to define treatment effectiveness.

The outcome was assessed at the last available follow-up visit.

Death occurring more than 12 months after documentation of treatment effectiveness was not considered treatment failure and was analyzed as a separate outcome.

Relapse was evaluated as a post-response outcome in patients who achieved treatment effectiveness, after discontinuation of immunosuppressive therapy and during ongoing immunosuppressive treatment. Relapse was defined as radiological progression, manifested by an increase in the thickness of the periaortic retroperitoneal mass or recurrence of hydronephrosis, accompanied by a concomitant rise in inflammatory markers.

Statistical analyses were performed using IBM SPSS Statistics version 29.0. Data distribution was assessed using the Shapiro-Wilk test. Continuous variables were presented as median (Q1-Q3), as the majority of variables were non-normally distributed. For variables with a limited number of observations, data were summarized as median (range). Continuous variables were compared using the Mann-Whitney U test. Categorical variables were presented as absolute numbers and percentages and were compared using the chi-square test of independence.

All statistical tests were two-sided.

Due to the retrospective nature of the study, analyses were conducted using available-case analysis without imputation of missing data. The number of patients included in each analysis varied depending on data availability.

No sensitivity analyses were performed.

A two-sided p-value < 0.05 was considered statistically significant.

Logistic regression analysis was performed to identify factors associated with treatment effectiveness, defined as a composite of clinical, laboratory, and radiological improvement. A forward stepwise logistic regression model was applied including baseline hydronephrosis, treatment type and baseline CRP levels as independent variables.

Additional univariable logistic regression analyses were performed for treatment type, hydronephrosis, CRP, ESR and serum creatinine levels as well as for baseline periaortic mass thickness.

Odds ratios (ORs) with 95% confidence intervals (CIs) were reported. Regression analyses were considered exploratory due to the limited sample size.

No formal sample size calculation was performed due to the retrospective nature of the study.

Potential sources of bias include selection bias and confounding by indication due to the non-randomized treatment allocation.

The study protocol was reviewed by the Local Bioethics Committee, which determined that formal ethical approval was not required due to the retrospective and non-interventional nature of the study.

## Results

### Study population

A total of 32 patients with retroperitoneal fibrosis treated between 2010 and 2025 were initially identified. Three patients were excluded from the analysis: two due to RPF secondary to malignancy and one due to delayed initiation of immunosuppressive therapy caused by a skin abscess, followed by loss to follow-up.

Histopathological evaluation was available in 9 patients. Biopsy of the retroperitoneal mass was performed in 6 patients and was diagnostic in 5 cases, confirming RPF, while 1 biopsy was non-diagnostic. In 3 patients, histopathological examination of other organs, including salivary glands or bile ducts, established a diagnosis of IgG4-RD and RPF was therefore classified as IgG4-related. In an additional 3 patients, granulomatosis with polyangiitis (GPA) was diagnosed, and RPF was classified as secondary to connective tissue disease. In the remaining patients, IRF was diagnosed based on typical imaging findings and exclusion of alternative etiologies.

Ultimately, 29 patients with idiopathic or secondary retroperitoneal fibrosis were included in the final analysis. Idiopathic RPF was diagnosed in 23 of 29 patients, while secondary RPF was identified in 6 of 29 patients, including 3 cases associated with IgG4-RD and 3 cases associated with GPA. Of these 29 patients, 11 were assigned to the intensive treatment group and 18 to the less intensive treatment group.

### Baseline characteristics

Baseline demographic characteristics of the study population are summarized in Table 1.

**Table 1:** Baseline demographic characteristics

Variable	Total	Intensive treatment	Less intensive treatment
Age at diagnosis, years, *n* = 29	60 (52–63)	60 (49–63)	60 (54–63)
Male sex, *n* = 29	21 (72.4)	10 (90.9)	11 (61.1)
Body mass index (BMI), kg/m^2^, *n* = 25	27.0 (24.2–29.5)	25.9 (25.1–27.3)	27.5 (23.8–30.4)
Smoking, *n* = 28	9 (32.1)	5 (45.5)	4 (22.2)
*Type of retroperitoneal fibrosis*			
Idiopathic	23 (79.3)	7 (63.6)	16 (88.9)
IgG4-related	3 (10.3)	1 (9)	2 (11.1)
GPA-associated	3 (10.3)	3 (27.3)	0

Data are presented as median (Q1–Q3) or number (%), as appropriate

The median age at diagnosis was 60 years (52–63), and the majority of patients were male (21/29, 72.4%).

Body mass index (BMI) data were available for 25 patients, with a median BMI of 27.0 kg/m^2^ (24.2–29.5). Smoking status was available for 28 patients, of whom 9 (32.1%) were current smokers.

Baseline disease severity and organ involvement are presented in Table 2.

**Table 2:** Baseline disease severity

Variable	Total	Intensive treatment	Less intensive treatment	*p*-value
Hydronephrosis, (*n* = 29)	19 (65.5)	6 (54.5)	13 (72.2)	0.331^a^
Bilateral hydronephrosis, (*n* = 29)	8 (27.6)	3 (27.3)	5 (27.8)	0.976^a^
Ureteral stents required, (*n* = 28)	13 (46.4)	5 (45.5)	8 (47)	0.934^a^
CRP at baseline, mg/dL (*n* = 22)	1.10 (0.30–2.15)	1.40 (0.20–1.40)	1.0 (0.40–4.30)	0.479^b^
ESR at baseline, mm/h (*n* = 21)	23 (5–66)	33 (4–35)	20 (5–70)	0.911^b^
Serum creatinine at baseline, mg/dL (*n* = 25)	1.10 (0.8–1.45)	0.90 (0.80–1.20)	1.20 (1.00-1.50)	0.104^b^
Hemoglobin at baseline, g/dL (*n* = 28)	13.1 (11.9–15.2)	13.3 (11.6–15.2)	12.9 (12.3–15.2)	0.832^b^
Baseline thickness of periaortic mass, mm (*n* = 21)	11.0 (8.75-14.0)	10.0 (9.0–12.0)	11.0 (8.5–16.0)	0.881^b^

Data are presented as median (Q1–Q3) or number (%), as appropriate

^a^Pearson Chi-Square

^b^Mann–Whitney *U* test

Hydronephrosis was present in 19 of 29 patients (65.5%), including bilateral hydronephrosis in 8 patients (27.6%). Ureteral stents were placed in 13 patients.

Importantly, no statistically significant differences were observed between the treatment groups with regard to baseline demographic characteristics, inflammatory markers, presence of hydronephrosis, or baseline thickness of the periaortic retroperitoneal mass.

Predictors of treatment response.

In the forward stepwise regression analysis (*n* = 18), baseline hydronephrosis was the only variable retained in the final model and was associated with higher odds of treatment response (OR = 15.0, 95% CI 1.2-185.2).

In the univariable logistic regression, none of the evaluated baseline variables were significantly associated with treatment effectiveness (Table 3).

**Table 3:** Univariable logistic regression analysis of treatment response

Variable	OR	95% CI	*p*-value
Treatment type	2.67	0.41–17.17	0.302
Hydronephrosis baseline	5.42	0.88–33.36	0.069
CRP baseline (mg/dL)	1.12	0.81–1.54	0.507
ESR baseline (mm/h)	1.01	0.98–1.03	0.603
Creatinine baseline (mg/dL)	1.61	0.38–6.77	0.516
Mass thickness baseline	1.04	0.84–1.30	0.706

Similarly, treatment type was not significantly associated with treatment effectiveness (OR = 2.67, 95% CI 0.41–17.17).

### Treatment effectiveness

Treatment effectiveness according to the predefined composite endpoint is summarized in Table 4. Overall, effective treatment was achieved in 17 of 25 evaluable patients (68.0%).

**Table 4:** Treatment effectiveness and outcomes

Variable	Total	Intensive treatment	Less intensive treatment	*p*-value
Overall treatment effectiveness (*n* = 25)	17 (68)	8 (80)	9 (60)	0.294^a^
Clinical improvement (*n* = 26)	22 (84.6)	10 (100)	12 (75)	0.086^a^
Laboratory response (*n* = 25)	22 (88)	9 (90)	13 (86.6)	0.802^a^
Radiological response (*n* = 25)	19 (76)	8 (88.9)	11 (68.8)	0.258^a^
Relative reduction in mass thickness, %, (*n* = 16)	41.15 (24.45–53.95)	52.30 (30.30-63.35)	37.50 (17.60–50.00)	0.173^b^
Resolution of hydronephrosis (*n* = 16)	12 (75)	5 (83.3)	7 (70)	0.551^a^
Time to ureteral stents (JJ) removal, months (*n* = 6)∗	17.50 (5.0–68.0)	26.0 (18.0–68.0)	6.0 (5.0–17.0)	0.050^b^
Treatment adverse events (*n* = 29)	9 (31)	3 (27.3)	6 (33.3)	0.732^a^
Follow-up duration, months (*n* = 28)	27.00 (11.00–82.00)	85.00 (45.00-120.00)	16.50 (7.50–31.00)	0.005^b^

^a^ Pearson Chi-Square

^b^ Mann–Whitney U test

Data are presented as median (Q1–Q3) or number (%), as appropriate

*For time to ureteral stents (JJ) removal, data are presented as median (range) due to the limited number of observations

Clinical improvement was observed in 22 of 26 patients (84.6%), laboratory response in 22 of 25 patients (88.0%), and radiological response among 25 evaluable patients was observed in 19 cases (76%). Hydronephrosis resolved in 12 patients (75%).

Overall treatment effectiveness was observed across both treatment strategies, with no significant evidence of additional benefit associated with escalation to intensive intravenous immunosuppression. (80% vs. 60%, *p* = 0.294). Moreover, no statistically significant differences between groups were observed with regard to individual components of the composite endpoint.

### Radiological and renal outcomes

The median interval between baseline and follow-up computed tomography examinations was 14 months (11–29.5 months).

The median thickness of the periaortic retroperitoneal mass decreased from 11.0 mm (8.75–14.0) at baseline to 6.75 mm (4.0–9.75) on follow-up imaging. The median relative reduction in mass thickness was 41.2% (24.45–53.95).

The magnitude of mass reduction did not differ significantly between groups (Table 4).

Radiological response was defined as a ≥20% retroperitoneal mass reduction and/or resolution of hydronephrosis on follow-up imaging. Consequently, radiological response was observed in 19 patients, whereas mass reduction was confirmed in 16 patients.

Renal outcomes were assessed by comparing peak serum creatinine levels observed during follow-up with serum creatinine values at last observation. Median peak serum creatinine levels during follow-up were 1.50 mg/dL (1.10–2.20) in the overall cohort and did not differ significantly between treatment groups (intensive treatment group: median 1.70 mg/dL (1.20–4.05) vs. less intensive treatment group: median 1.50 mg/dL (1.00-1.88), (*p* = 0.268).

At last observation, median serum creatinine levels decreased to 1.20 mg/dL (0.90–1.40) in the overall cohort, indicating improvement in renal function over time.

No statistically significant differences were observed between the intensive and less intensive treatment groups, with median serum creatinine levels of 1.20 mg/dL (1.10–1.30) and 1.20 mg/dL (0.80–1.40), respectively; (*p* = 0.402).

Removal of ureteral stents was possible in 6 of 11 patients (54.5%), with 3 patients in each group.

Among patients requiring ureteral stenting, the time to ureteral stent removal was shorter in the less intensive treatment group, with a difference approaching statistical significance (Table 4).

### Glucocorticoid exposure

Characteristics of systemic glucocorticoid therapy are summarized in Table 5. Patients in the intensive treatment group received significantly higher initial glucocorticoid doses compared with those in the less intensive treatment group, with median doses of 60 mg/day (60.00-72.50) and 40 mg/day (22.50–60.00), (*p* = 0.002).

**Table 5:** Glucocorticoid treatment characteristics

Variable	Total	Intensive treatment	Less intensive treatment	p-value
Initial glucocorticoid dose, mg/day (*n* = 27)	60.00 (30.00–60.00)	60.00 (60.00-72.50)	40.00 (22.50–60.00)	0.002^a^
Glucocorticoid discontinuation (*n* = 23)	18 (78.3)	8 (72.7)	10 (83.3)	0.538^b^
Time to glucocorticoid discontinuation, months (*n* = 18)	15.0 (12.0–24.0)	16.50 (10.50-21.25)	15.0 (12.0-25.50)	0.592^a^
Re-initiation of glucocorticoids after withdrawal (*n* = 22)	12 (54.5)	7 (87.5)	5 (35.7)	0.019^b^

Data are presented as median (Q1–Q3) or number (%), as appropriate

^a^Mann–Whitney U test

^b^ Pearson Chi-Square

No statistically significant differences were observed between groups in the proportion of patients in whom steroids were discontinued or in the time to glucocorticoid discontinuation.

In contrast, re-initiation of systemic glucocorticoid therapy after prior withdrawal occurred significantly more frequently in the intensive treatment group.

### Mortality, relapse, and treatment safety

The median duration of follow-up for the entire cohort was 27 months (11–82). Patients in the intensive treatment group had a significantly longer follow-up compared with patients in the less intensive treatment group, with median follow-up durations of 85 months (45–120) and 16.5 months (7.5–31).

Among the 17 patients who achieved treatment effectiveness, relapse could be evaluated in 15, as they had a sufficiently long follow-up after confirmation of treatment efficacy. Disease relapse occurred in 2 of these 15 patients. Both relapses developed 12 months after remission induced by cyclophosphamide, during maintenance immunosuppressive therapy with methotrexate.

During follow-up, four deaths were recorded, all occurring in patients receiving intensive treatment. However, this observation should be interpreted with caution, as patients in the intensive treatment group had a substantially longer follow-up.

Among the four patients, who died during follow-up, the cause of death remained unknown in two cases. One patient treated with cyclophosphamide died due to severe pneumonia, and one previously treated with rituximab had infective endocarditis prior to death.

In an exploratory analysis comparing patients who died during follow-up and those with good prognosis (Table 6), patients who died demonstrated a lower thickness of the periaortic retroperitoneal mass on follow-up imaging, with a difference approaching statistical significance, with median values of 2.0 mm (0.0–2.0) and 7.5 mm (4.75-10.0), (*p* = 0.050).

**Table 6:** Mortality analysis (exploratory)

Variable	Total*n* = 29	Good prognosis*n* = 25	Death*n* = 4	p-value
ESR after treatment, mm/h (*n* = 23)	8.00 (4.00–18.00)	7.00 (3.00–18.00)	34.00 (12.50–91.50)	0.046^a^
Baseline mass thickness, mm (*n* = 21)	11.00 (8.75-14.00)	10.75 (8.63-15.00)	12.00 (12.00–12.00)	0.454^a^
Follow-up mass thickness, mm (*n* = 20)	6.75 (0.0–16.00)	7.50 (2.00–16.00)	2.00 (0.0–4.0)	0.050^a^
Follow-up duration, months (*n* = 28)	27.0 (3.0-–168.0)	30.0 (3.0–168.0)	24.0 (10.0–84.0)	0.911^a^

Data are presented as median (Q1-Q3)

^a^ Mann–Whitney U test

Notably, patients who died during follow-up had significantly higher ESR values at follow-up compared with patients with good prognosis, with median ESR values of 34.0 mm/h (12.5–91.5) and 8.0 mm/h (4.0–18.0), (*p* = 0.046).

The substantially longer follow-up in the intensive treatment group represents an important source of bias and may have influenced the observed differences in outcomes, particularly relapse and mortality.

Adverse events leading to treatment discontinuation were observed in 9 of 29 patients (31.0%). The most common adverse event was recurrent urinary tract infection, which occurred in 3 patients (1 in the intensive treatment group and 2 in the less intensive treatment group).

One patient treated with CYC developed severe pneumonia during therapy and subsequently died. The remaining side effects occurred exclusively in patients from the less intensive treatment group. No statistically significant differences in the frequency of adverse events were observed between treatment groups (Table 4).

## Discussion

Despite decades of clinical experience, retroperitoneal fibrosis remains a disease without established standardized treatment guidelines. Its rarity, marked clinical heterogeneity, and the broad range of immunosuppressive agents used in clinical practice have resulted in most published data being derived from small, retrospective patient cohorts, thereby limiting the ability to directly compare the effectiveness of different therapeutic strategies [17, 18, 20]. 

This study provides real-world comparative data on different immunosuppressive treatment strategies in RPF, addressing the lack of direct comparative evidence in the current literature.

In a recent systematic review with meta-analysis, Steimer et al. showed no statistically significant differences in the effectiveness of RPF treatment between individual groups of immunosuppressive drugs [21]. 

In addition, treatment response in RPF has been defined inconsistently across studies. While many reports emphasize radiological endpoints, such as regression of the periaortic mass or resolution of hydronephrosis, clinical and laboratory responses are variably incorporated into outcome definitions. As a result, meaningful comparisons between studies remain challenging [9, 10]. 

Most available studies focus on the effectiveness of a single immunosuppressive agent, while direct comparisons between different immunosuppressive therapies are rarely performed, presumably as a consequence of small and heterogeneous patient groups [9, 13]. 

Importantly, previous studies have demonstrated that adding an immunosuppressive agent to corticosteroids in the treatment of RPF allows for a reduction in steroid dose with a similar or higher treatment response rate [22, 23]. 

Only two studies have directly compared the efficacy of cyclophosphamide with other immunosuppressive agents in the treatment of RPF. Marcolongo et al. reported comparable effectiveness of azathioprine and cyclophosphamide, whereas Binder et al. demonstrated superiority of CYC compared with other immunosuppressive strategies in achieving treatment response in chronic periaortitis [11, 14]. To our knowledge, no studies have directly compared the efficacy of rituximab with other immunosuppressive agents in the therapy of RPF.

In the literature, substantial evidence supports the effectiveness of rituximab in IgG4-RD, which may also manifest as secondary RPF. Hammitzsch et al. demonstrated the efficacy and safety of rituximab in IgG4-RD, while two recent review studies confirmed high response rates, particularly in patients with severe or refractory disease manifestations [24–26]. 

In this retrospective, single-center observational study, we evaluated the effectiveness and safety of two different immunosuppressive treatment strategies in patients with idiopathic and secondary forms of RPF associated with immune-mediated diseases. The principal finding of our study is that overall treatment effectiveness, defined as a composite endpoint incorporating clinical, laboratory, and radiological responses, did not differ significantly between patients treated with intensive intravenous regimens and those receiving less intensive oral immunosuppressive therapy. Despite comparable efficacy rates, relevant differences between treatment strategies were observed with regard to glucocorticoid exposure, urological outcomes, and mortality.

Overall treatment effectiveness was achieved in 68% of evaluable patients, which is consistent with response rates reported in previous studies [7, 13]. Clinical and laboratory improvement were observed more frequently than radiological response, a finding in line with prior observations that regression of fibrotic tissue may lag behind symptomatic and biochemical improvement [16]. The median relative reduction in periaortic mass thickness exceeding 40% in the present cohort surpasses the threshold commonly considered clinically meaningful in the literature and supports the validity of our radiological response criteria [9, 19]. Importantly, no significant differences were observed between treatment groups for any individual component of the composite endpoint, suggesting that both treatment approaches may achieve comparable disease control in a subset of patients.

In this study, no consistent predictors of treatment effectiveness were identified. Treatment type was not associated with improved outcomes.

Baseline hydronephrosis was associated with treatment effectiveness in the stepwise logistic regression model; however, hydronephrosis was included as part of the composite endpoint of radiological improvement, which may introduce circular bias and therefore this association should be interpreted with caution and not considered a reliable independent predictor of treatment response.

The absence of significant predictors may reflect the heterogeneity of the study population and limited statistical power.

Improvement of renal function represents the most clinically relevant outcome in RPF, given the high prevalence of ureteral obstruction and hydronephrosis at diagnosis. This is particularly relevant given that the presence of hydronephrosis is associated with a poorer prognosis [27]. 

In our cohort, renal function improved during observation comparably in both treatment groups, as reflected by decreasing serum creatinine levels during follow-up. Similarly, resolution of ureteral obstruction occurred at comparable rates between groups. These findings suggest that both intensive and less intensive immunosuppressive strategies may be effective in preventing irreversible renal damage when appropriately combined with urological interventions.

In this study, immunosuppressive therapy was selected based on the individual decision of the treating physician. As a result, patients were not randomly assigned to treatment groups, introducing the possibility of confounding by indication, which represents a limitation of this retrospective study. The absence of clear superiority of cyclophosphamide- or rituximab-based regimens over less intensive oral therapies in terms of treatment effectiveness warrants careful consideration. Patients in the intensive treatment group received significantly higher initial glucocorticoid doses and were more likely to require re-initiation of steroids after withdrawal. This likely reflects more refractory disease course rather than inferior efficacy of intensive immunosuppression therapy. These observations highlight the complexity of treatment selection in RPF, where therapeutic decisions are frequently guided by disease severity, renal involvement, and physician experience in the absence of standardized treatment guidelines.

Relapse remains a clinically relevant issue in RPF, as the optimal duration of immunosuppressive therapy and the appropriate length of patient monitoring have not been clearly established, and treatment discontinuation may be associated with an increased risk of disease recurrence. Previous studies have demonstrated that prolonged low-dose glucocorticoid therapy is beneficial in maintaining disease remission and reducing the risk of relapse [27]. 

In the present cohort relapse occurred in a minority of patients and was observed exclusively in the intensive treatment group. This finding should be interpreted with caution given the small number of evaluable patients and the substantially longer follow-up duration in the intensive treatment group. Nevertheless, it underscores the relapsing nature of RPF and the need for long-term monitoring after confirming the apparent therapeutic response.

Mortality was observed during follow-up only among patients treated with cyclophosphamide or rituximab. However, given the retrospective design of the study, the limited number of events, and the longer follow-up duration in the intensive treatment group, mortality should not be considered as a direct treatment-related effect. Exploratory analyses revealed that patients who died during follow-up had higher inflammatory marker levels despite marked regression of the retroperitoneal mass, suggesting that radiological improvement alone may not fully capture ongoing disease activity or prognosis of RPF.

Adverse events affected nearly one-third of patients, with no significant differences in frequency between groups. Recurrent urinary tract infections were the most common complications observed. This represents a major clinical challenge in patients with hydronephrosis following urological interventions, as immunosuppressive therapy substantially increases the risk of the infectious complications, while patients with hydronephrosis often require more intensive treatment [17, 28]. 

Most publications on retroperitoneal fibrosis published in Rheumatology International have been limited to case reports or small case series, with relatively limited data regarding different immunosuppressive treatment strategies. One of the larger previously published cohorts by Liu et al. demonstrated favorable outcomes with immunosuppressive therapy and highlighted the potential role of long-term immunosuppression in preventing relapses [29]. Our study expands these observations by evaluating treatment outcomes in a real-world retrospective cohort and directly comparing intensive intravenous and less intensive immunosuppressive strategies in RPF using a composite clinical, laboratory, and radiological outcome definition.

Our findings suggest that escalation to intensive intravenous immunosuppressive therapy does not necessarily translate into superior clinical outcomes in RPF.

Several limitations of this study should be acknowledged. Its retrospective design, single-center setting, and relatively small patient cohorts limit the ability to draw definitive conclusions regarding comparative treatment effectiveness. Additionally, heterogeneity in immunosuppressive regimens and differences in follow-up duration between treatment groups may have influenced the observed outcomes. Nevertheless, given the rarity of RPF, our findings provide valuable real-world data on treatment efficacy, renal outcomes, and long-term disease course. Furthermore, imaging assessments, although performed using a predefined measurement approach, were not independently validated.

Prospective, multicenter studies are needed to establish standardized definitions of treatment response and to optimize long-term management strategies for this rare and heterogeneous disease.

## Conclusion

In this real-world cohort of patients with RPF, intensive intravenous immunosuppressive therapy was not associated with superior treatment effectiveness compared with less intensive oral regimens. Comparable clinical, laboratory, radiological, and renal outcomes were observed between groups.

These findings underscore the importance of individualized approach to treatment selection, guided by disease severity and patient- specific factors. Further prospective studies are needed to define optimal management strategies in RPF.

## Declarations

### AI statement

AI-assisted tools were used exclusively for language editing purposes (OpenAI, GPT-5.5, March 2026). The authors are fully responsible for the study design, data analysis, and the content of the manuscript. No part of this report was copied or generated using AI tools.
